# Trends in CT examination utilization in the emergency department during and after the COVID-19 pandemic

**DOI:** 10.1186/s12880-024-01457-4

**Published:** 2024-10-21

**Authors:** Felix Kempter, Tobias Heye, Jan Vosshenrich, Benjamin Ceresa, Dominik Jäschke

**Affiliations:** Department of Radiology, University Hospital Basel, Petersgraben 4, Basel, 4031 Switzerland

**Keywords:** Radiology, COVID-19, Computed tomography, Emergency service, Health services misuse

## Abstract

**Background:**

The increasing use of CT imaging in emergency departments, despite efforts of reducing low-value imaging, is not fully understood, especially during and after the COVID-19 pandemic. The aim of this study was to investigate the impact of COVID-19 pandemic related measures on trends and volume in CT examinations requested in the emergency department.

**Methods:**

CT examinations of the head, chest, and/or abdomen-pelvis (*n* = 161,008), and chest radiographs (*n* = 113,240) performed at our tertiary care hospital between 01/2014 and 12/2023 were retrospectively analyzed. CT examinations (head, chest, abdomen, dual-region and polytrauma) and chest radiographs requested by the emergency department during (03/2020-03/2022) and after the COVID-19 pandemic (04/2022-12/2023) were compared to a pre-pandemic control period (02/2018-02/2020). Analyses included CT examinations per emergency department visit, and prediction models based on pre-pandemic trends and inpatient data. A regular expressions text search algorithm determined the most common clinical questions.

**Results:**

The usage of dual-region and chest CT examinations were higher during (+ 116,4% and + 115.8%, respectively; *p* < .001) and after the COVID-19 pandemic (+ 88,4% and + 70.7%, respectively; *p* < .001), compared to the control period. Chest radiograph usage decreased (-54.1% and − 36.4%, respectively; *p* < .001). The post-pandemic overall CT examination rate per emergency department visit increased by 4.7%. The prediction model underestimated (*p* < .001) the growth (dual-region CT: 22.3%, chest CT: 26.7%, chest radiographs: -30.4%), and the rise (*p* < .001) was higher compared to inpatient data (dual-region CT: 54.8%, chest CT: 52.0%, CR: -32.3%). Post-pandemic, the number of clinical questions to rule out “pulmonary infiltrates”, “abdominal pain” and “infection focus” increased up to 235.7% compared to the control period.

**Conclusions:**

Following the COVID-19 pandemic, chest CT and dual-region CT usage in the emergency department experienced a disproportionate and sustained surge compared to pre-pandemic growth.

## Background

The utilization of computed tomography (CT) in emergency departments has revolutionized assessing acute conditions with speed and precision [1, 2]. However, disproportionate surges in CT imaging compared to the increase in emergency visits have been described [3–6]. Initiatives like “Choosing Wisely”, highlight a growing focus on reducing unnecessary imaging in emergency departments [7]. More in depth analyses, for example by Kjelle et al. [8] revealed that a considerable share of diagnostic imaging remains to be low-value imaging – defined as having little or no patient benefit, wasting resources, and increasing healthcare costs [9]. Despite various proposed explanations for the increased use of CT in emergency departments, the underlying reasons remain unclear [4–6, 10]. Furthermore, a universally accepted definition for the excessive utilization of imaging is not yet established [11, 12]. Recently, changes in CT examination volumes [13–15], particularly an increase in chest CT usage during the COVID-19 pandemic [15–18], were noted. Higher chest CT examination volumes persisted post-pandemically [16].

The aim of our study was to examine the impact of the COVID-19 pandemic on CT examination volumes in the emergency department in a university hospital setting in Switzerland during and after COVID-19 pandemic related measures, anticipating that changes encountered during the COVID-19 pandemic would subsequently influence examination volumes in the post-pandemic period. Particularly, trends in volumes of the five most requested CT examinations were analyzed to investigate shifts towards the more frequent use of multi-region CTs instead of single-region CTs as an indicator of imaging overuse.

## Methods

The local ethics committee of northwestern and central Switzerland (project ID 2022 − 01016) approved this study. Written informed consent was waived. There is no conflict of interest, and no financial support was received for this study.

### Study sample

**Fig. 1:**
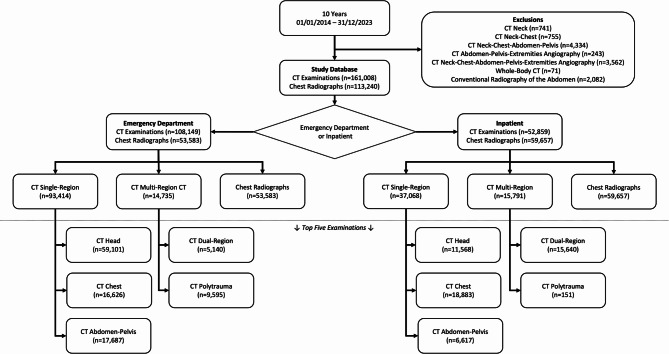
Flowchart of the study. CT = computed tomography

Our retrospective, single-center study included all CT examinations (*n* = 161,008) including the head, chest, and/or abdomen performed in a university hospital center in Switzerland over a 10-year period (January 1, 2014, and December 31, 2023) without further preselection. CT examinations requested by the emergency department are reviewed by radiologists before being conducted. The cohort’s median patient age was 55 years (Interquartile Range (IQR): 27–83; 53.7% men). Additionally, 113,240 chest radiographs (median patient age: 54 years (IQR: 27–82); 59.3% men) were included. A detailed flowchart of the study sample is provided in Fig. 1.

Reports of the examinations written in German as reporting language, and examination metadata (date, modality, examination type) were stored in a database for analysis.

### Study periods

Four specific time intervals within a 10-year study-period were defined: (1) historic period (HIST), January 1, 2014 to January 31, 2018, representing a historical dataset for comparisons and predictive model development, (2) control period (CTRL): February 1, 2018 to February 29, 2020, representing recent pre-pandemic trends and matching the duration of the COVID-19 pandemic period to serve as a reference for the assessment of pandemic and post-pandemic changes, (3) COVID-19 pandemic period (COV), March 1, 2020 to March 31, 2022, aligning with the duration of the COVID-19 pandemic in Switzerland, and (4) post-pandemic period (POST): April 1, 2022 to December 31, 2023, after pandemic-related measures were lifted by the Swiss Federal Office of Public Health [19].

### CT body regions

CT examinations were divided into single-region CTs, covering only the head, chest, or abdomen-pelvis, respectively, and multi-region CTs, either chest-abdomen-pelvis, or polytrauma scans. These were also the five most frequently requested CT examinations in the emergency department (head, *n* = 59,101; chest, *n* = 16,626; abdomen-pelvis, *n* = 17,687; chest-abdomen-pelvis (= dual-region), *n* = 5,140; and polytrauma, *n* = 9,595).

### CT scanners

Four CT scanners were utilized simultaneously during the study period. Between January and March 2020, a Siemens Somatom Definition AS + was replaced with a Siemens Somatom Force. From April to June 2021, a Siemens Somatom Definition Flash was replaced with another Siemens Somatom Force (Dual Source). Two other scanners, a Siemens Somatom Definition Edge and a Siemens Somatom Definition AS, have been in operation since 2014.

### Text processing

To investigate the frequency of specific clinical questions from CT examination referrals, regular expressions with a rule-based syntax for text data processing were used [20]. The clinical question field of every CT examination was analyzed for the conditions listed in Table 1, including grammatical and orthographical variations. Conditions represented a modified selection from an existing study [21] to align with institutional considerations of clinical importance and prevalence.

**Table 1:** Medical conditions

Medical Conditions	
Abdominal pain, Abscess, Aneurysm, Appendicitis, Aspiration, Atelectasis, Bleeding, Cholecystitis, Cholelithiasis, Cholangitis, COPD, COVID-19, Dissection, Diverticulitis, Emphysema, Empyema, Foreign body, Fracture, Free fluid, Fungal disease, Hemothorax, Hernia, Ileus, Infiltrate, Infection focus, Ischemia, Pancreatitis, Perforation, Pleural effusion, Pneumothorax, Pulmonary embolism, Pulmonary edema, Sarcoidosis, Trauma, Tuberculosis, Tumor, Urolithiasis	

List of conditions that were searched for in clinical questions section of CT requests using a text search algorithm based on regular expressions.

### Data analysis

Computations were performed in commercially available software (Tableau Desktop 2022.3 and JMP 17.2). Descriptive statistics were calculated overall, and for distinct examination types across time intervals with monthly aggregated data. Following Shapiro-Wilk and Anderson-Darling tests to assess data distribution; computations to assess differences between time intervals and trends, with the control period (CTRL) serving as the reference for comparison with COV and POST periods. Tests included ANOVA and Tukey-Kramer HSD for normally distributed data, and Kruskal-Wallis and Dunn’s tests for non-normally distributed data. Furthermore, linear regression analysis was used to determine relationships between imaging volumes and months/years. *P*-values < 0.05 were considered indicative of statistically significant differences.

Emergency department visits were tracked from February 1, 2018, to December 31, 2023. The data on emergency department visits are derived from complex control systems, with reliable records available only since 2018 and could not be traced back to 2014. However, radiological imaging data, recorded using a different methodology, allowed tracking back to 2014. Relative monthly differences and average CT imaging volumes per visit were calculated and compared between CTRL with COV and POST periods. Predictions in imaging volumes until December 31, 2023, were computed based on pre-pandemic data from HIST and CTRL periods. Projected volumes were compared with actual volumes in the POST period using averaged monthly aggregated data.

## Results

### Examination volume trends overall and body region

**Fig. 2:**
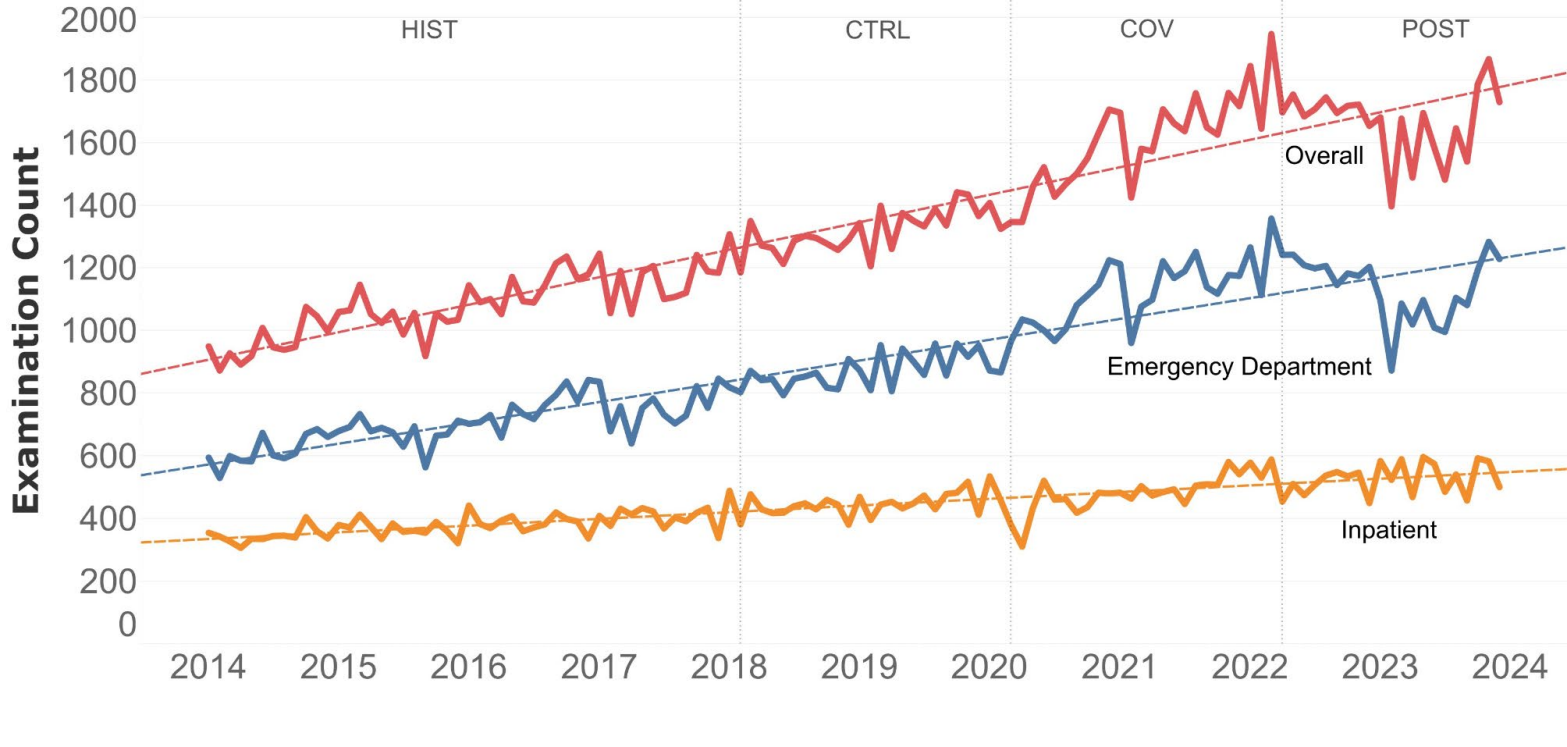
Monthly CT examination counts (January 2014 - December 2023) in the emergency department, for inpatient, and overall. Graphs include trending lines to illustrate changes. HIST = historic period, CTRL = control period, COV = COVID-19 pandemic period, POST = post-pandemic period

**Fig. 3:**
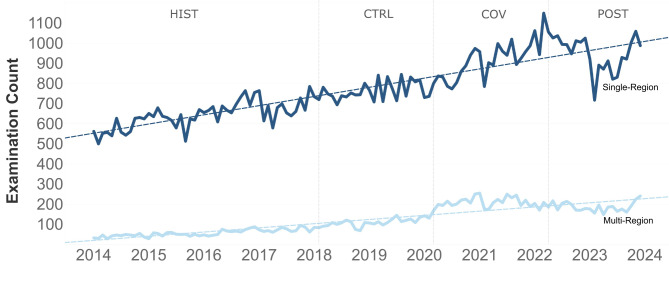
Monthly CT examination counts (January 2014 - December 2023) for emergency department subgroups (single- and multi-region CT exams). Graphs include trending lines. HIST = historic period, CTRL = control period, COV = COVID-19 pandemic period, POST = post-pandemic period

Between January 2014 and December 2023, the average monthly CT examination at our institution increased by 87.7 mean examination counts monthly overall (*p* < .001, R^2^ = 0.88). Mean examination counts increased by 66.0 per month for CT scans requested by the emergency department (*p* < .001, R^2^ = 0.84), and 21.6 per month for CT scans requested for inpatients (*p* < .001, R^2^ = 0.72). Trends are visualized in Fig. 2. Monthly single body-region CT scans requested by the emergency department increased by 45.3 (*p* < .001; R²=0.80) and monthly multi-region CTs increased by 20.7 (*p* < .001; R²=0.81). Trends for single-region and multi-region CTs are visualized in Fig. 3.

Dual-region CT scans experienced the highest relative increase in monthly volume (17.8%; 7.6 scans; *p* < .001, R²=0.74), followed by CT polytrauma (16.4%; 13.1 scans; *p* < .001, R²=0.79), CT chest (12.6%; 17.4 scans; *p* = .003, R²=0.53), CT head (4.7%; 23.3 scans; *p* < .001, R²=0.69), and CT abdomen-pelvis examinations (3.1%; 4.6 scans; *p* < .001, R²=0.35). Overall, there were no significant patient age-related trends observed.

### Examinations volumes compared by study periods

Mean overall monthly CT examination counts increased from HIST to CTRL (mean: 701 ± 79 vs. 872 ± 52; *p* < .001), and further increased to 1,124 ± 102 during COV (*p* < .001), persisting into POST (1,137 ± 104; *p* < .001). Data is summarized in Table 2.

For dual-region CT scans, an increase during COV (*p* < .001) and POST (*p* < .001) periods was found, with monthly volumes rising by 41.2 ± 2.7 (+ 116.4%) and 31.3 ± 2.8 (+ 88.4%), compared to the CTRL period. Similarly, chest CT and polytrauma CT volumes experienced a substantial increase. Chest CT volumes increased by 134.0 ± 10.6 (+ 115.8%) and 75.3 ± 11.1 (+ 70.7%) during COV and POST, respectively (both *p* < .001). Polytrauma CT monthly examinations increased by 58.1 ± 5.0 (+ 77.9%) and 45.6 ± 5.3 (+ 61.1%), respectively (both *p* < .001). Monthly head CT examination counts increased only in the post-pandemic period (+ 111.7 ± 16.3; +22.6%; *p* < .001). Abdomen-pelvis CT counts did not show statistically significant changes during COV (*p* = .21) and POST (*p* = .96), compared to the CTRL period.

For chest radiographs, monthly examination volumes decreased by 42.9 ± 5.8 (-54.1%) during COV (*p* < .001). Lower volumes persisted in the POST period, with 26.6 ± 6.1 (-36.4%) fewer exams per month compared to CTRL (*p* < .001).

**Table 2:** Examinations volumes compared by study periods

	Historic Period (01/2014–01/2018) (01/2014–01/2018)	Control Period (02/2018–02/2020)	COVID-19 Period (03/2020–03/2022)	Post-Pandemic Period (04/2022–12/2023)	Overall (01/2014–12/2023)
**CT Overall**					
Count (% of total)	34,366 (100%)	21,800 (100%)	28,100 (100%)	23,883 (100%)	108,149 (100%)
Mean Monthly Count (± SD)	701.3 (± 78.5)*	872.0 (± 52.4)	1,124.0 (± 101.7)*	1,137.3 (± 103.8)*	901.2 (± 209.4)
Difference to Control-Period (%)	-19.6%	n/a	28.9%	30.4%	n/a
Median age (IQR)	60 (37–83)	60 (37–82)	60 (37–83)	61 (38–83)	60 (35–85)
Sex (Men | Women)	52.4% | 47.6%	52.7% | 47.3%	53.9% | 46.2%	53.0% | 47.0%	52.9% | 47.1%
**CT Single-region**					
Count (% of total)	31,539 (91.8%)	19,049 (87.4%)	22,868 (81.4%)	19,958 (83.6%)	93,414 (86.4%)
Mean Monthly Count (± SD)	643.7 (± 66.6)*	762.0 (± 45.7)	914.7 (± 93.1)*	950.4 (± 88.2)*	778.5 (± 148.1)
Difference to Control-Period (%)	-15.5%	n/a	20.1%	24.7%	n/a
**CT Multi-region**					
Count (% of total)	2,827 (8.2%)	2,751 (12.6%)	5,232 (18.6%)	3,925 (16.4%)	14,735 (13.6%)
Mean Monthly Count (± SD)	57.7 (± 16.7)*	110.0 (± 19.1)	209.3 (± 24.9)*	186.9 (± 23.6)*	122.8 (± 66.9)
Difference to Control-Period (%)	-47.5%	n/a	90.2%	69.9%	n/a
**CT Head**					
Count (% of total)	20,947 (61.0%)	12,366 (56.8%)	13,054 (46.5%)	12,734 (53.4%)	59,101 (54.7%)
Mean Monthly Count (± SD)	427.5 (± 44.9)*	494.6 (± 31.4)	522.2 (± 74.1)	606.4 (± 50.4)*	492.5 (± 82.2)
Difference to Control-Period (%)	13.6%	n/a	5.6%	22.6%	n/a
**CT Chest**					
Count (% of total)	4,132 (12.0%)	2,663 (12.2%)	6,013 (21.4%)	3,818 (16.0%)	16,626 (15.3%)
Mean Monthly Count (± SD)	84.3 (± 15.5)*	106.5 (± 16.1)	240.5 (± 53.9)*	181.8 (± 31.3)*	138.6 (± 69.6)
Difference to Control-Period (%)	20.8%	n/a	125.8%	70.7%	n/a
**CT Abdomen-Pelvis**					
Count (% of total)	6,460 (18.8%)	4,020 (18.4%)	3,801 (13.4%)	3,406 (14.2%)	17,687 (16.3%)
Mean Monthly Count (± SD)	131.8 (± 19.6)*	160.8 (± 17.2)	152.0 (± 21.1)	162.2 (± 15.6)	147.4 (± 22.9)
Difference to Control-Period (%)	18.0%	n/a	-5.5%	0.9%	n/a
**CT Dual-Region**					
Count (% of Total)	938 (2.7%)	886 (4.0%)	1,915 (6.8%)	1,401 (5.9%)	5,140 (4.8%)
Mean Monthly Count (± SD)	19.1 (± 6.3)*	35.4 (± 5.1)	76.6 (± 12.7)*	66.7 (± 8.8)*	42.8 (± 25.5)
Difference to Control-Period (%)	-46.0%	n/a	116.1%	88.3%	n/a
**CT Polytrauma**					
Count (% of total)	1,889 (5.5%)	1,865 (8.6%)	3,317 (11.8%)	2,524 (10.6%)	9,595 (8.9%)
Mean Monthly Count (± SD)	38.6 (± 13.2)*	74.6 (± 16.4)	132.7 (± 18.0)*	120.2 (± 19.1)*	80.0 (± 42.8)
Difference to Control-Period (%)	-48.3%	n/a	77.9%	61.1%	n/a
**Chest Radiographs**					
Count	28,174	12,747	5,849	3,813	53,583
Mean Monthly Count (± SD)	576.0 (± 70.6)*	509.9 (± 54.6)	234.0 (± 68.7)*	324.4 (± 50.2)*	446.5 (± 154.1)
Difference to Control-Period (%)	12.8%	n/a	-54.1%	-36.4%	n/a
Median age (IQR)	61 (39–84)	60 (38–82)	60 (38–82)	60 (38–82)	62 (39–85)
Sex (Men | Women)	53.8% | 46.2%	55.4% | 44.6%	56.6% | 43.4%	55.9% | 44.1%	54.8% | 45.2%

Absolute and relative numbers of CT examinations, and average monthly examination counts in the emergency department overall, and stratified by time period. Note - Percentages of total are computed per time period and rounded to the first decimal place. Statistically significant differences (*p* < .05) compared to the control-period are marked with an asterisk.

### Comparison with emergency department visits

Average monthly emergency department visits increased from 4,157.8 ± 118.6 in CTRL to 4,330.7 ± 1,067.7 (+ 4.2%, *p* = 1.00) in COV and to 4,429.8 ± 224.6 (+ 6.5%, *p* = .001) in POST. Overall CT examination rates grew from 21.0 CT examinations per 100 emergecy department visits in the CTRL period to 27.1 (*p* < .001) during the pandemic and stayed elevated with 25.7 (*p* < .001) post-pandemic. The same was true for dual-region CT growing from 0.9 examinations per 100 emergency department visits in CTRL to 1.9 (+ 111.1%, *p* < .001) in COV and 1.5 (+ 66.7%, *p* < .001) in POST, chest CT from 2.6 examinations per 100 emergency department visits in CTRL to 5.8 (+ 113.1%, *p* < .001) in COV and 4.1 (+ 57.7%, *p* < .001) in POST, polytrauma CT from 1.8 examinations per 100 emergency department visits in CTRL to 3.2 (+ 77.8%, *p* < .001) in COV and 2.7 (+ 50.0%, *p* < .001) in POST (COV: +77.8%, *p* < .001; POST: +50.0%, *p* < .001) and head CT examination rates from 11.9 examinations per 100 emergency department visits in CTRL to 12.6 (+ 5.9%, *p* = .002) in COV and 13.7 (+ 15.1%, *p* < .001) in POST. Chest radiograph examination rates decreased during the same periods from 12.3 examinations per 100 emergency department visits in CTRL to 5.5 (-55.3%, *p* < .001) in COV and 7.3 (-40.7%, *p* < .001) in POST. Data is summarized in Table 3.

**Table 3:** Comparison with Emergency Department visits

	Historic Period (01/2014–01/2018) (01/2014–01/2018)	Control Period (02/2018–02/2020)	COVID-19 Period (03/2020–03/2022)	Post-Pandemic Period (04/2022–12/2023)	Overall (01/2014–12/2023)
**Emergency Department Visits**					
Count	n/a	103,945	108,268	93,026	305,239
Mean Monthly Count (± SD)	n/a	4,157.8 (± 118.6)	4,330.7 (± 1,067.7)	4,429.8 (± 224.6)	4,299.1 (± 650.1)
Difference to Control-Period (%)	n/a	n/a	4.2%	6.5%	n/a
**CT Overall**					
Utilization rate per visit (% [± SD])	n/a	21.0% (± 1.1%)	27.1% (± 5.3%)*	25.7% (± 1.9%)*	24.5% (± 4.3%)
Difference to Control-Period (%)	n/a	n/a	29.0%	22.4%	n/a
**CT Single-region**					
Utilization rate per visit (% [± SD])	n/a	18.3% (± 1.0%)	22.0% (± 4.3%)*	21.4% (± 1.7%)*	20.5% (± 3.2%)
Difference to Control-Period (%)	n/a	n/a	20.2%	16.9%	n/a
**CT Multi-region**					
Utilization rate per visit (% [± SD])	n/a	2.6% (± 0.4%)	5.0% (± 1.1%)*	4.2% (± 0.5%)*	4.0% (± 1.3%)
Difference to Control-Period (%)	n/a	n/a	92.3%	61.5%	n/a
**CT Head**					
Utilization rate per visit (% [± SD])	n/a	11.9% (± 0.8%)	12.6% (± 2.6%)*	13.7% (± 1.0%)*	12.7% (± 1.8%)
Difference to Control-Period (%)	n/a	n/a	5.9%	15.1%	n/a
**CT Chest**					
Utilization rate per visit (% [± SD])	n/a	2.6% (± 0.4%)	5.8% (± 1.7%)*	4.1% (± 0.6%)*	4.1% (± 1.7%)
Difference to Control-Period (%)	n/a	n/a	113.1%	57.7%	n/a
**CT Abdomen-Pelvis**					
Utilization rate per visit (% [± SD])	n/a	3.9% (± 0.4%)	3.7% (± 0.8%)	3.7% (± 0.3%)	3.7% (± 0.6%)
Difference to Control-Period (%)	n/a	n/a	-5.1%	-5.1%	n/a
**CT Dual-Region**					
Utilization rate per visit (% [± SD])	n/a	0.9% (± 0.1%)	1.9% (± 0.5%)*	1.5% (± 0.2%)*	1.4% (± 0.5%)
Difference to Control-Period (%)	n/a	n/a	111.1%	66.7%	n/a
**CT Polytrauma**					
Utilization rate per visit (% [± SD])	n/a	1.8% (± 0.4%)	3.2% (± 0.7%)*	2.7% (± 0.4%)*	2.6% (± 0.8%)
Difference to Control-Period (%)	n/a	n/a	77.8%	50.0%	n/a
**Chest Radiographs**					
Utilization rate per visit (% [± SD])	n/a	12.3% (± 1.3%)	5.5% (± 1.5%)*	7.3% (± 1.2%)*	8.4% (± 3.3%)
Difference to Control-Period (%)	n/a	n/a	-55.3%	-40.7%	n/a

Number of patient visits in the emergency department, monthly averages, and CT utilization rate overall, and stratified by time period.

Note - Statistically significant differences (*p* < .05) compared to the control-period are marked with an asterisk.

### Comparison with predicted volumes

**Fig. 4:**
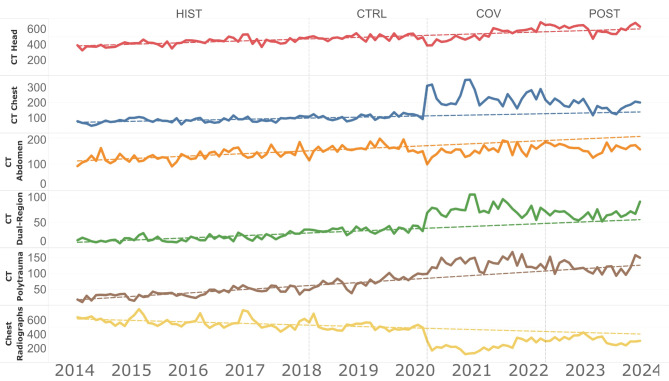
Monthly CT examination counts (January 2014 - December 2023) for the five most frequent examinations requested by the emergency department, included forecast trending lines (dashed lines). Projections are based on pre-pandemic periods (January 2014 - February 2020) and extrapolated through December 2023. HIST = historic period, CTRL = control period, COV = COVID-19 pandemic period, POST = post-pandemic period, CT = computed tomography

Monthly chest CT examinations showed the highest relative difference to volumes predicted based on previous growth, with actual average monthly exam counts being 26.7% or 48.5 ± 6.9 higher than expected (*p* < .001). Similarly, monthly dual-region CT examinations volumes were 22.3% or 14.8 ± 2.0 exams higher (*p* < .001), and head CT volumes were 3.9% or 23.4 ± 11.3 exams higher than predicted (*p* = .045). In contrast, monthly chest radiograph volumes were 30.4%, or 98.5 ± 11.2 exams lower than expected (*p* < .001). The same was true for abdomen-pelvis CT examinations, with 22.4%, or 36.3 ± 3.6 fewer monthly scans than expected (*p* < .001). Monthly polytrauma CT examinations counts did differ from predicted volumes (*p* = .50). Figure 4 provides a comprehensive overview of the actual monthly examinations counts during the post-pandemic (POST) period, compared to the predicted volumes based on data from the historic and control periods.

### Comparison with inpatient data

The increase in examination volumes requested by the emergency department was higher than those for inpatients. The highest difference was seen for dual-region CT examinations, with an increase in monthly CT requests by the emergency department being 54.8 ± 7.7% higher than for inpatients in the same time period (*p* < .001). Similarly, chest CT requests by the emergency department were 52.0 ± 10.1% higher than the observed increase for inpatient CT requests (*p* < .001). The decrease in chest radiograph usage was more pronounced in the emergency department, than for inpatients (32.3 ± 3.0%; *p* < .001). In contrast, the decrease in CT abdomen-pelvis requests was higher for inpatients, than for the emergency department (12.0 ± 4.1%; *p* = .02). Trends in ordering patterns for head CT scans did not differ (*p* = .30). Examination volume trends overall and body regions for inpatient data are summarized in Table 4.

**Table 4:** Comparison with Inpatient Data

	Historic Period (01/2014–01/2018) (01/2014–01/2018)	Control Period (02/2018–02/2020)	COVID-19 Period (03/2020–03/2022)	Post-Pandemic Period (04/2022–12/2023)	Overall (01/2014–12/2023)
**CT Overall**					
Count (% of total)	18,535 (100%)	11,169 (100%)	12,086 (100%)	11,069 (100%)	52,859 (100%)
Mean Monthly Count (± SD)	378.3 (± 37.4)	446.8 (± 36.9)	483.4 (± 61.7)*	527.1 (± 49.0)*	440.5 (± 73.0)
Difference to Control-Period (%)	-15.3%	n/a	8.2%	18.0%	n/a
Median age (IQR)	48.5 (25–72)	51 (28–74)	50.5 (27–74)	51 (28–74)	50 (25–75)
Sex (Men | Women)	53.9% | 46.1%	53.5% | 46.5%	54.5% | 45.5%	53.4% | 46.6%	53.8% | 46.2%
**CT Single-region**					
Count (% of total)	12,978 (70.0%)	7,977 (71.4%)	8,620 (71.3%)	7,493 (67.7%)	37,068 (70.1%)
Mean Monthly Count (± SD)	264.9 (± 28.1)	319.1 (± 28.8)	344.8 (± 45.8)*	356.8 (± 35.3)*	308.9 (± 51.1)
Difference to Control-Period (%)	-17.0%	n/a	8.1%	11.8%	n/a
**CT Multi-region**					
Count (% of total)	5,557 (30.0%)	3,192 (28.6%)	3,466 (28.7%)	3,576 (32.3%)	15,791 (29.9%)
Mean Monthly Count (± SD)	113.4 (± 13.3)	127.7 (± 16.1)	138.6 (± 19.6)	170.3 (± 22.8)*	131.6 (± 26.5)
Difference to Control-Period (%)	-11.2%	n/a	8.6%	33.4%	n/a
**CT Head**					
Count (% of total)	4,378 (23.6%)	2,356 (21.1%)	2,561 (21.2%)	2,273 (20.5%)	11,568 (21.9%)
Mean Monthly Count (± SD)	89.3 (± 12.6)	94.2 (± 9.7)	102.4 (± 9.8)	108.2 (± 18.6)*	96.4 (± 14.7)
Difference to Control-Period (%)	-5.2%	n/a	8.7%	14.9%	n/a
**CT Chest**					
Count (% of total)	6,013 (32.4%)	4,083 (36.6%)	4,715 (39.0%)	4,072 (36.8%)	18,883 (35.7%)
Mean Monthly Count (± SD)	122.7 (± 19.3)	163.3 (± 18.4)	188.6 (± 35.2)*	193.9 (± 23.8)*	157.4 (± 38.8)
Difference to Control-Period (%)	-24.9%	n/a	15.5%	18.7%	n/a
**CT Abdomen-Pelvis**					
Count (% of total)	2,587 (14.0%)	1,538 (13.8%)	1,344 (11.1%)	1,148 (10.4%)	6,617 (12.5%)
Mean Monthly Count (± SD)	52.8 (± 10.0)	61.5 (± 11.3)	53.8 (± 9.5)*	54.7 (± 8.3)	55.1 (± 10.4)
Difference to Control-Period (%)	-14.2%	n/a	-12.6%	-11.1%	n/a
**CT Dual-Region**					
Count (% of Total)	5,520 (29.8%)	3,161 (28.3%)	3,416 (28.3%)	3,543 (32.0%)	15,640 (29.6%)
Mean Monthly Count (± SD)	112.7 (± 13.3)	126.4 (± 15.6)	136.6 (± 19.5)*	168.7 (± 22.3)*	130.3 (± 26.1)
Difference to Control-Period (%)	-10.9%	n/a	8.1%	33.4%	n/a
**CT Polytrauma**					
Count (% of total)	37 (0.2%)	31 (0.3%)	50 (0.4%)	33 (0.3%)	151 (0.3%)
Mean Monthly Count (± SD)	1.4 (± 0.9)	1.7 (± 1.0)	2.4 (± 1.3)	2.2 (± 1.0)	1.9 (± 1.1)
Difference to Control-Period (%)	-17.6%	n/a	41.2%	29.4%	n/a
**Chest Radiographs**					
Count	24,941	12,677	11,826	10,213	59,657
Mean Monthly Count (± SD)	509.0 (± 42.9)	507.1 (± 34.0)	473.0 (± 34.3)*	486.3 (± 34.6)	497.1 (± 40.4)
Difference to Control-Period (%)	0.4%	n/a	-6.7%	-4.1%	n/a
Median age (IQR)	49 (25–74)	49 (25–74)	49 (25–74)	49 (24–74)	51 (25–76)
Sex (Men | Women)	53.8% | 46.2%	55.4% | 44.6%	56.6% | 43.4%	55.9% | 44.1%	54.8% | 45.2%

Absolute numbers, monthly averages, and relative changes in CT examination counts in inpatients stratified by time period. Note – Percentages of total are computed per time period and rounded to the first decimal place. Statistically significant differences (*p* < .05) compared to the control-period are marked with an asterisk.

### Text processing analysis

Only clinical questions occurring ≥ 20% of CT requests (“pulmonary embolism”, “pulmonary infiltrates”, “infection focus”) and “abdominal pain” due to its strong relative increase from CTRL to COV (3.4%) and POST (4.9%) were included. These occurred in 72.7% of dual-region CT and 86.8% of chest CT orders from the emergency department. All four clinical questions increased from CTRL to COV and POST periods for dual-region CT scans and chest CT scans (excluding “abdominal pain”), respectively (*p* < .001; Table 5).

**Fig. 5:**
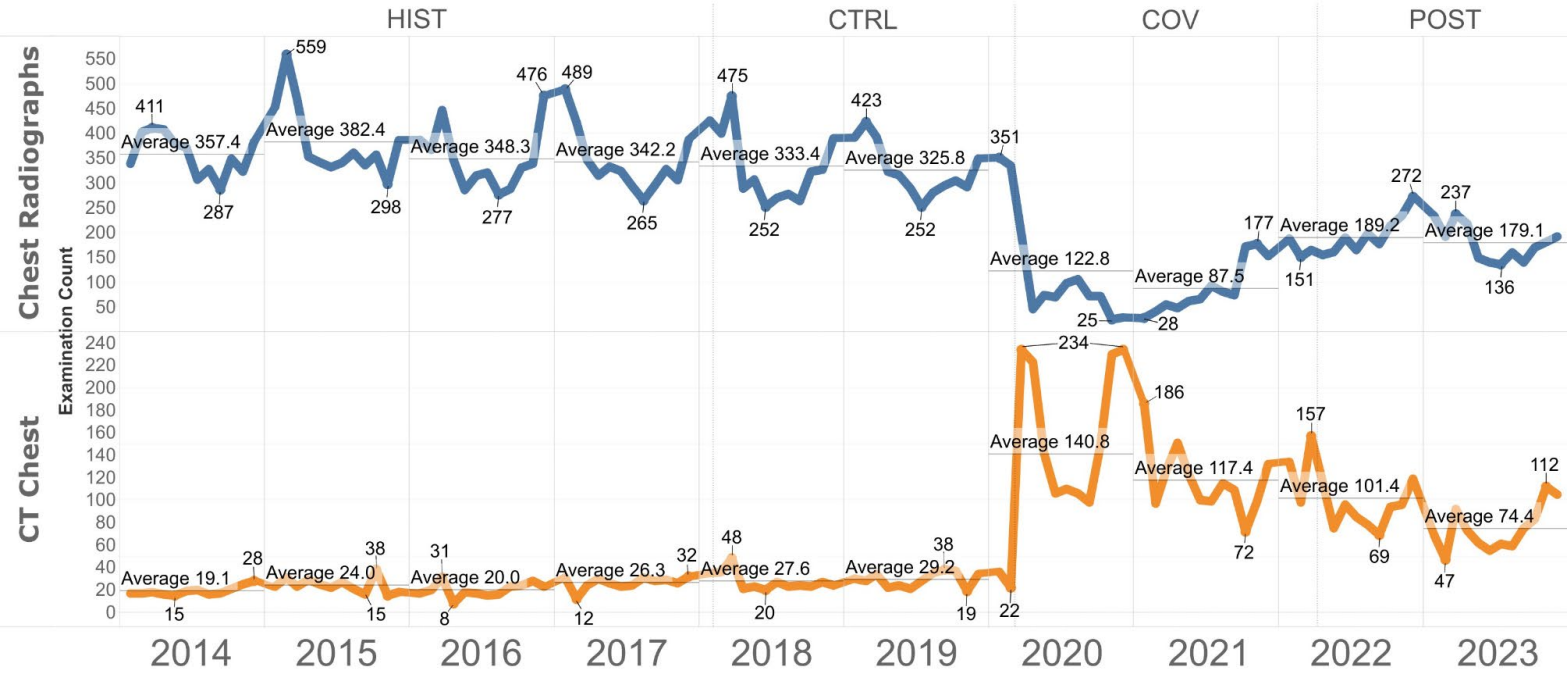
Monthly chest radiograph and chest CT examinations counts for “pulmonary infiltrates” (January 2014 - December 2023), including annual means, minimums, and maximums. Please note disappearing seasonality in chest radiograph utilization during and post COVID-19 periods compared to pre-pandemic periods. CT = computed tomography, HIST = historic period, CTRL = control period, COV = COVID-19 pandemic period, POST = post-pandemic period

For comparison, the occurrence of “pulmonary infiltrates” in chest radiograph requests decreased for COV and did not completely reverse for POST (both *p* < .001; CTRL: 8,171 exams, 64.10%; COV: 2,341 exams, 40.02%; POST: 3,915 exams, 57.46%). Trends in monthly examination counts are visualized in Fig. 5. As visually discernible, the pre-pandemic seasonal pattern of chest radiograph utilization for “pulmonary infiltrates” was disrupted during the COVID-19 pandemic. This pattern has not reverted to its prior form in the post-pandemic period.

**Table 5:** Text processing analysis

	Historic Period (01/2014–01/2018) (01/2014–01/2018)	Control Period (02/2018–02/2020)	COVID-19 Period (03/2020–03/2022)	Post-Pandemic Period (04/2022–12/2023)	Overall (01/2014–12/2023)
**CT Chest**					
**Pulmonary Embolism**					
Count (% of total of CT Chest)	2,841 (68.8%)	1,875 (70.4%)	3,160 (52.6%)	2,495 (65.3%)	10,371 (62.4%)
Mean Monthly Count (± SD)	58.0 (± 12.6)*	75.0 (± 12.8)	126.4 (± 38.2)*	118.8 (± 24.3)*	86.4 (± 37.0)
Difference to Control-Period (%)	-22.7%	n/a	68.5%	58.4%	n/a
**Pulmonary Infiltrate**					
Count (% of total of CT Chest)	1,108 (26.8%)	704 (26.4%)	3,430 (57.0%)	1,721 (45.1%)	6,963 (41.9%)
Mean Monthly Count (± SD)	22.6 (± 6.4)*	28.2 (± 7.3)	137.2 (± 48.1)*	82.0 (± 20.6)*	58.0 (± 4.7)
Difference to Control-Period (%)	-19.9%	n/a	386.5%	190.8%	n/a
**Infection Focus**					
Count (% of total of CT Chest)	122 (3.0%)	141 (5.3%)	685 (11.4%)	395 (10.3%)	1,343 (8.1%)
Mean Monthly Count (± SD)	2.7 (± 1.5)*	5.6 (± 2.3)	27.4 (± 12.5)*	18.8 (± 5.7)*	11.5 (± 11.9)
Difference to Control-Period (%)	-51.8%	n/a	389.3%	235.7%	n/a
**Abdominal Pain**					
Count (% of total of CT Chest)	23 (0.6%)	12 (0.5%)	41 (0.7%)	50 (1.3%)	126 (0.8%)
Mean Monthly Count (± SD)	1.3 (± 0.6)	1.5 (± 1.1)	2.0 (± 1.0)	2.6 (± 1.1)	1.9 (± 1.1)
Difference to Control-Period (%)	-13.3%	n/a	33.3%	73.3%	n/a
**CT Dual-Region**					
**Pulmonary Embolism**					
Count (% of total CT Dual Region)	180 (19.2%)	227 (25.6%)	418 (21.8%)	293 (20.9%)	1,118 (21.8%)
Mean Monthly Count (± SD)	3.8 (± 2.3)*	9.1 (± 2.5)	16.7 (± 7.3)*	14.0 (± 4.1)*	9.5 (± 6.7)
Difference to Control-Period (%)	-58.2%	n/a	83.5%	53.8%	n/a
**Pulmonary Infiltrate**					
Count (% of total of CT Dual Region)	168 (17.9%)	144 (16.3%)	467 (24.4%)	317 (22.6%)	1,096 (21.3%)
Mean Monthly Count (± SD)	3.5 (± 1.8)*	5.8 (± 2.7)	18.7 (± 7.2)*	15.1 (± 5.6)*	9.2 (± 7.7)
Difference to Control-Period (%)	-39.7%	n/a	222.4%	160.3%	n/a
**Infection Focus**					
Count (% of total of CT Dual Region)	323 (34.4%)	371 (41.9%)	1,061 (55.4%)	836 (59.7%)	2,591 (50.4%)
Mean Monthly Count (± SD)	6.6 (± 3.8)*	14.8 (± 4.0)	42.4 (± 8.3)*	39.8 (± 6.1)*	21.6 (± 16.8)
Difference to Control-Period (%)	-55.4%	n/a	186.5%	168.9%	n/a
**Abdominal Pain**					
Count (% of total of CT Dual Region)	66 (7.0%)	66 (7.4%)	207 (10.8%)	172 (12.3%)	511 (9.9%)
Mean Monthly Count (± SD)	1.9 (± 1.2)*	2.9 (± 1.3)	8.3 (± 2.3)*	8.2 (± 3.2)*	5.0 (± 3.6)
Difference to Control-Period (%)	-34.5%	n/a	186.2%	182.8%	n/a

Absolute and relative numbers of CT examinations requested by the emergency department stratified by time period and clinical question. Note – Percentages of total are computed per time period and rounded to the first decimal place. Statistically significant differences (*p* < .05) compared to the control-period are marked with an asterisk.

## Discussion

The aim of this study was to explore the effects of the COVID-19 pandemic on the utilization of CT examinations in the emergency department, focusing on the five most frequently requested examination types before, during, and after pandemic-related measures being in effect. We observed a notable increase in the usage of dual-region CT (chest-abdomen-pelvis) (+ 88.4%) and chest CT scans (+ 70.7%) in the post-pandemic period compared to pre-pandemic levels. Simultaneously, chest radiograph volumes decreased significantly (-36.4%). Comparative data analyses confirmed rising CT examination volumes to vastly exceed (i) the accompanying increase in emergency department patient visits, (ii) predictions based on pre-pandemic data, and (iii) increase in examination volumes requested for inpatient over the same time period.

The increase in CT examination volumes in the emergency department has already been reported prior to the COVID-19 pandemic [3–6]. Nevertheless, the use of most imaging tests dropped with the beginning of the pandemic [15, 18, 22–26]. Despite most studies reporting an absolute decrease in CT examination volumes during the initial lockdown [18, 23–25], the relative use of CT per emergency department visit increased [14]. Contrary observations in absolute numbers, where limited to particular geographic regions, for example in northern Italy [22], potentially linked to high COVID-19 case volumes in early 2020 [27]. Over the extended course of the pandemic, absolute CT utilization increased [15–17], which aligns with our findings. Particularly, the usage of chest CT scans surged during the pandemic [14–18] and persisted into the post-pandemic era, as reported by Arıkan et al. [16]

Despite not being recommended as a primary screening method or for mildly symptomatic cases of COVID-19 [28], CT imaging provides rapid and comprehensive insights into pulmonary involvement and is crucial to streamline patient management. Moreover, CT provided faster results than RT-PCR tests during peak pandemic periods [15, 29]. These factors are thought to represent the main reasons for surging chest CT utilization in emergency departments amidst the pandemic [15–17].

Our study is not limited to chest CT examination frequency analyses, but also investigated clinical questions from CT request forms. Mean monthly examination requests to rule out “pulmonary infiltrates” increased from 28.2 before the pandemic to 82.0 in the post-pandemic period. Simultaneously, the use of chest radiographs for “pulmonary infiltrate” decreased in our study, matching reported findings in decreases of general usage of chest radiographs by Arikan et al. [16] since chest radiographs have limited sensitivity in detecting COVID-19 [30]. Before the pandemic, the use of chest radiographs for “pulmonary infiltrates” displayed a seasonal trend, disrupted during the pandemic [20]. Our results indicate no visible return to the pre-pandemic pattern in the post-pandemic period. The persistent decrease in chest radiograph usage coupled with the increase in chest CT usage for “pulmonary infiltrates” and the non-return of seasonal pattern of chest radiographs, however, indicate a paradigm-shift in the usage of imaging tests following the COVID-19 pandemic.

Even before pandemic-attributed variations in the usage of diagnostic imaging, an increased use of multi-region CT examinations in emergency department over single-region CTs has been reported, most notably for chest-abdomen-pelvis studies [6] and whole-body CT [31]. The specific reasons remain to be fully understood [4–6, 10]. Our study noted similar a disproportionate surge in dual-region CT scans and an increase in nonspecific clinical questions, such as “abdominal pain” and “infection focus”, during and after COVID-19. Along with the mentioned investigations, our results support the hypothesis that diagnostic uncertainty among referring clinicians is a possible factor for the increase of dual-region CT examinations [6, 32]. In the local medical context, the term “infection focus” is used to describe any type of infection, including those that progress to sepsis. Identifying the septic focus can be challenging for ED physicians due to limited medical history at the initial presentation. While microbiological analysis of blood and fluids is crucial for isolating pathogens, it takes time for cultures, whereas imaging results are available faster [33] and the causal treatment can be adjusted immediately [34, 35]. International sepsis guidelines do not provide specific recommendations regarding the use of CT imaging for identifying the source of infection [36], although it is highly beneficial with a high sensitivity [37] and could be performed in patients with unclear clinical infection [35]. Conversely, it is important to acknowledge that Schleder et al. (2017) reported the absence of an infectious focus in 37% of dual-region CT scans, confirmed the diagnosis in 28%, altered the diagnosis in 35%, and led to a change in therapy in 30% of cases [35]. Nonetheless, these findings do not account for the 168.9% increase in identified “infection focus” observed after COVID-19 when compared to before the COVID-19 pandemic.

In general, CT imaging provides rapid and precise diagnostics [1, 2], potentially allowing to discharge low-risk patients sooner and save costs in some cases [38, 39]. However, the growing discussion regarding the overutilization of imaging, particularly of CT examinations, highlights implications for growing healthcare costs, radiation exposure, workload, and environmental impact [40–42]. While recent summaries of low-value imaging did not note shifts from radiographs to single- or multi-region CT during COVID-19 in particular [8] our observations indicate this might be a non-negligible factor when reviewing how paradigms in clinical practice and CT usage have shifted post-pandemically, building upon already substantial growth rates in CT usage before COVID-19 at our institution. Brandsæter et al. have suggested that it becomes more convenient for referring physicians to rely on imaging results, rather than clinical assessment, and the greater willingness to accept false positive results than to miss a rare or unusual cause of a patient’s symptoms [43]. The introduction of advanced CT scanners can also increase imaging volumes [44]. However, our data shows no direct correlation, and no CT scanner specifically optimized for chest imaging was installed during the study period. Device-related effects would likely have faded within 6–12 months after installation, especially as emergency department referrals are often unaware of scanner upgrades. Another possible explanation for the observed shift might be rooted in the development of suboptimal habits or less extensive training among residents and emergency physicians during the pandemic. Nevertheless, also radiologists have a role in driving low-value imaging, since accepting referrals may sometimes be more straightforward and timesaving, than discussing requests of examinations with questionable benefit with the referring physician [43]. Incorporating adequate review measures by radiologists into patient care can improve imaging safety and cost-efficiency, including the provision of feedback to ordering physicians [45]. These end-to-end feedback mechanisms are crucial to facilitate learning and improvement, and limit low-value imaging [46]. Without their consistent use, the healthcare system risks entering a self-sustaining loop that may ultimately prove unsustainable from both economic and environmental perspectives.

This study has several limitations. Firstly, the retrospective nature and single-center study design limits the generalizability, given globally varying responses to the pandemic responses, healthcare systems, and cultural factors. Secondly, the observed pandemic shift from chest radiographs to chest CTs in this study may in part be attributed to the institutional adoption of CT as the standard imaging test for suspected COVID-19 pneumonia between 2020 and 2022. Nevertheless, this practice was discontinued after COVID-19 restrictions were lifted. Thirdly, only the clinical questions section of the reports was examined. In a subsequent step, the report’s actual diagnosis could be analyzed to assess the precision of referring physician’s clinical assessment. Finally, while a substantial sample size was employed to validate the algorithm’s accuracy, data mislabeling might have occurred in some instances.

## Conclusions

Following the COVID-19 pandemic, chest CT and dual-region CT usage in the emergency department experienced a disproportionate and sustained surge compared to pre-pandemic growth. Post-pandemic surges in chest CT and dual-region CT usage in the emergency department, alongside broader clinical questions, suggest a rise in low-value imaging, warranting for further investigation of the causes, appropriateness, and effects on patient safety and costs.

## Data Availability

Data generated or analyzed during the study are available from the corresponding author by request.

## Abbreviations

COV: COVID-19 pandemic period
COVID-19: Coronavirus disease of 2019
CT: Computed tomography
CTRL: Control-period
HIST: Historic period
IQR: Interquartile range
POST: Post-restriction period
